# Understanding tensions and identifying clinician agreement on improvements to early-stage chronic kidney disease monitoring in primary care: a qualitative study

**DOI:** 10.1136/bmjopen-2015-010337

**Published:** 2016-03-17

**Authors:** Rosemary Simmonds, Julie Evans, Gene Feder, Tom Blakeman, Dan Lasserson, Elizabeth Murray, Kristina Bennert, Louise Locock, Jeremy Horwood

**Affiliations:** 1 Centre for Academic Primary Care, School of Social and Community Medicine, University of Bristol, UK; 2 Nuffield Department of Primary Care Health Sciences, University of Oxford, UK; 3 National Institute for Health Research Collaboration for Leadership in Applied Health Research and Care (NIHR CLAHRC) Greater Manchester, Centre for Primary Care, Institute of Population Health, University of Manchester, Manchester, UK; 4 NIHR Oxford Biomedical Research Centre, John Radcliffe Hospital, Oxford, UK; 5 e-Health Unit, Research Department of Primary Care and Population Health, UCL, UK; 6 National Institute for Health Research Collaboration for Leadership in Applied Health Research and Care (NIHR CLAHRC) West at University Hospitals Bristol NHS Foundation Trust

**Keywords:** PRIMARY CARE, QUALITATIVE RESEARCH

## Abstract

**Objectives:**

Since 2006, general practitioners (GPs) in England, UK, have been incentivised to keep a register and monitor patients with chronic kidney disease (CKD) stages 3–5. Despite tensions and debate around the merit of this activity, there has been little qualitative research exploring clinician perspectives on monitoring early-stage CKD in primary care. This study aimed to examine and understand a range of different healthcare professional views and experiences of identification and monitoring in primary care of early-stage CKD, in particular stage 3.

**Design:**

Qualitative design using semistructured interviews.

**Setting:**

National Health Service (NHS) settings across primary and secondary care in South West England, UK.

**Participants:**

25 clinicians: 16 GPs, 3 practice nurses, 4 renal consultants and 2 public health physicians.

**Results:**

We identified two related overarching themes of dissonance and consonance in clinician perspectives on early-stage CKD monitoring in primary care. Clinician dissonance around clinical guidelines for CKD monitoring emanated from different interpretations of CKD and different philosophies of healthcare and moral decision-making. Clinician consonance centred on the need for greater understanding of renal decline and increasing proteinuria testing to reduce overdiagnosis and identify those patients who were at risk of progression and further morbidity and who would benefit from early intervention. Clinicians recommended adopting a holistic approach for patients with CKD representing a barometer of overall health.

**Conclusions:**

The introduction of new National Institute for Health and Care Excellence (NICE) CKD guidelines in 2014, which focus the meaning and purpose of CKD monitoring by increased proteinuria testing and assessment of risk, may help to resolve some of the ethical and moral tensions clinicians expressed regarding the overmedicalisation of patients with a CKD diagnosis.

**Strengths and limitations of this study**

- This qualitative study explores how clinicians responsible for patients with chronic kidney disease (CKD) view guidance on identification and monitoring of early-stage CKD.
- Our purposive sampling strategy recruited several strata of professional groups caring for a range of patients.
- The qualitative data provide a deeper understanding of how clinicians manage complex ethical and philosophical tensions in their efforts to provide good patient care.
- The qualitative findings from our purposive sample are not intended to be representative but highlight important insights.

## Introduction

Since the international adoption of the Kidney Disease Outcomes Quality Initiative (KDOQI) in 2002 (modified in 2012),^1^ increasing numbers of people have received a diagnosis of chronic kidney disease (CKD). The stimulus for the 2002 framework was the absence of an agreed definition and classification of kidney disease and evidence that people were experiencing avoidable harm through late presentation.^2^ In 2010, the prevalence of CKD was described as having reached ‘epidemic proportions'^3^ having increased from 0.3% in 2005 to 3.9% in 2009.^4^ Between 2009 and 2010, CKD was thought to have accounted for 1.3% of the total UK healthcare budget with the majority of patients being managed within primary care.^5^

UK national guidance for the early identification and management of adults with CKD published in 2008 by the National Institute for Health and Care Excellence (NICE) recommends regular follow-up testing of all patients with CKD from stages 3–5. CKD stages are based on estimated glomerular filtration rate (eGFR) representing the level of kidney function.^6^ Since 2006, general practitioners (GPs) in England have been incentivised to keep a register of patients with CKD stages 3–5 through the Quality and Outcomes Framework (QOF). The rationale for CKD monitoring was for early identification of decline in renal function and management of cardiovascular risk.^2^ However, since the introduction of the CKD QOF register, there have been tensions around the management of people with CKD. In diagnosing CKD, the use of single eGFR measurements and ‘inappropriate use’ of microalbuminuria^7^ are thought to have contributed to missed diagnoses and overclassifications of CKD. It is argued that these problems have created increased GP workloads/use of health resources and ‘suboptimal management’ of patients in primary care.^4^ ^8^

Concerns and tensions around CKD as a new disease paradigm^7^ have been the subject of debate in some key biomedical journals over the last decade, particularly over stable early-stage CKD. Minor reductions in eGFR alone without indicators of glomerular damage may be considered an aspect of normal ageing and these issues have been articulated in the ‘too much medicine’, ‘bad medicine’^9^ and the ‘medicalisation of normal ageing’ debates.^10^ Ethical issues around labelling people with a disease, who may never progress, were thought to present a potential for harm to patients^10^ as were concerns around the accuracy of diagnostic tests for CKD and the overdiagnosis of people over 75 years being labelled with the disease.^11^ ^12^ The clinical significance of low eGFR in the elderly has raised considerable debate.^2^ Reflecting these concerns, some clinicians did not accept the CKD disease paradigm,^13^ preferring not to disclose early-stage CKD, particularly in older patients.^14^ There were also concerns about the possible harm to elderly patients in pursuing target-driven blood pressure reduction in CKD.^12^ The evidence for these targets was criticised for being drawn from trials that ‘often exclude the elderly and those with mild-to-moderate CKD’.^12^

To date, qualitative studies examining the management of CKD in primary care have highlighted a number of tensions that presented barriers to optimal management.^13^ ^15^ ^16^ There was uncertainty around the merits of disclosure of a CKD diagnosis, particularly in older people.^15^ ^16^ Primary care clinicians were also reluctant to use the label ‘CKD’ with their patients, preferring to use terminology deemed less anxiety provoking and centred around reassurance.^13^ ^15^ ^16^ However, while these strategies protected patients from the emotional impact of a CKD diagnosis, it also limited opportunities for discussion with patients about kidney health and self-care that could help protect them from further kidney damage. This study aimed to examine the views and experiences of range of clinicians and to explore in more depth the issues of tension previously identified, in particular how clinicians understand and define early-stage CKD in relation to current guidelines and how this impacts on their approach to disclosure and care.

## Methods

Semistructured interviews were conducted with primary care clinicians, renal consultants and public health physicians across South West England, UK.

### Participant sampling and recruitment

Purposive sampling was used to capture maximum variation in experience of clinicians with responsibility for patients with CKD, either individually or at population level. Thirteen general practices in Bristol were purposively sampled to include practices serving a range of deprived and affluent areas using the practice-level indices of multiple deprivation (IMD) scores,^17^ size of patient list, percentage of older people and number of patients on the CKD register. To investigate experiences of CKD identification and monitoring in primary care, GPs and practice nurses were purposively sampled for maximum variation across year of qualification and GP practice. In addition, to contextualise the findings, we opportunistically interviewed four renal consultants and two public health physicians regarding their views on CKD3 monitoring.

### Data collection

The majority of interviews were conducted at the clinician's place of work and four interviews were conducted over the telephone. All interviews were conducted by a non-clinical researcher (RS) and lasted between 30 and 60 min. Although a topic guide was used to focus the interviews, participants were able to speak freely about their experiences and raise topics not covered by the guide. The topic guide was informed by a review of relevant literature and suggestions from our multiprofessional study team and advisory group and modified as data analysis progressed. Topics included perceptions of early-stage CKD (stage 3), experiences of CKD diagnosis, management and monitoring and opinions of current guidelines.

With written informed consent, all interviews were audio-recorded and transcribed verbatim, anonymised and imported into NVivo V.10 qualitative software program to aid management and analysis of data.

### Data analysis

RS led the analysis using an inductive thematic approach^18^ involving a process of constant comparison between cases.^19^ Analysis began alongside data collection, with ideas from early analysis informing later data collection in an iterative process until data indicated saturation.^20^ Analysis of individual transcripts started with open coding grounded in the data. This generated an initial coding framework, which was added to and refined as new data were gathered. Codes were gradually built into broader categories through comparison across transcripts, and higher level recurring themes were developed. To enhance analysis and enable team discussion and interpretation, team members (RS and JH) independently coded transcripts; any discrepancies were discussed to achieve a coding consensus and maximise rigour. The data, disconfirming views, coding frameworks and development of higher order and final themes were discussed by the multidisciplinary research team KB, JE, GF and JH to ensure credibility and confirmability.^21^

## Results

A total of 25 clinicians were recruited and interviewed: 16 GPs, 3 practice nurses, 4 renal consultants and 2 public health physicians (table 1). Interviews were conducted between February and September 2014. Analysis led to the development of the key emergent themes outlined below. All initials of participants refer to pseudonyms.

**Table 1:** Participant characteristics

		Years since qualification			GP practice levels of deprivation*	
Participants	N=25	<5 years	5–9 years	10+ years	Deprived	Affluent
GPs	16	1	3	12	8	8
Nurses	3	0	0	3	3	0
		**Years in current role**				
		**<5 years**	**5–9 years**	**10+ years**		
Renal consultants	4	0	2	2	All levels of deprivation	
Public health physician	2	1	0	1	All levels of deprivation	

*GP practice levels of deprivation measured as Index of Multiple Deprivation (IMD) quintile from GP practice postcode.^17^

GP, general practitioner.

### Understanding the tensions: clinician dissonance around early-stage CKD monitoring

Cognitive dissonance^22^ refers to the discomfort experienced when an individual holds contradictory beliefs, ideas or values or is confronted by new information that conflicts with existing beliefs, ideas or values. Most GPs expressed initial discomfort about being incentivised through QOF to keep a register and monitor of patients with CKD stage 3, as it contradicted existing beliefs around the meaning and status of mild reduction in kidney function:

I think when it first came in as an idea…we all rolled our eyes and went ‘Oh my God’, you know? They're creating an illness that doesn't exist. (GPD)

The introduction of ‘CKD’ as a new disease construct conflicted with most GP professional values and personal understandings of general practice medicine. GPs referred to general practice medicine as primarily responding to patients' symptoms and explained how monitoring activities and screening programmes were changing the way they practised. GPs experienced dissonance between what they felt was their role as generalists in caring for individual patients and what was being recommended by English national guidelines^23^ and incentivised via QOF, at a population level.

People get confused between screening and actually picking disease up, because people present with symptoms, which is actually what most medicine is…this idea of bringing in back-door screening…you know, I think they should be honest about what they're actually trying to do and particularly when you're looking at harm to patients—increased anxiety, labelling…interesting areas for the next five years of discussion, but I suspect it will go down more and more—disease labelling, monitoring…that's where we'll go. (GPJ)

GPs also felt tension between their experiences of managing patients with impaired kidney function in general practice and the rationale for labelling patients with a disease. GPs felt that the introduction of CKD monitoring in primary care was driven by specialists informed by experience of advanced, progressive kidney disease which they perceived as rare in primary care. As a result, GPs felt that patients were being medicalised and potentially harmed by being given a chronic disease label that did not relate to significant renal pathology.

I don't think that the people who introduced this were actually aware of the natural history of 95% of eGFRs of 60…it was actually looked at from the wrong end of the telescope ie, you know, it was the specialist looking at the people that ended up with them with kidney failure who then assumed that…this was the usual situation as opposed to being aware, actually, that it doesn't end up as kidney failure out in the community…I expect GPs are probably more aware of monitoring eGFR levels, which is what we're really talking about here, rather than trying to label it as a disease. (GPC)

We medicalise everything…instead of normalising, we're making people ill…and we're putting labels on them which don't really benefit them at all most of the time…. and the patient rarely, rarely benefits…“It's not really an illness—it's a bit like being 5 foot 2”… for the vast majority of the patients that we see, it has no practical importance or relevance at all. (GPA)

A renal consultant also experienced dissonance around classifying ‘disease’ in patients where the eGFR levels could be considered ‘normal’ in relation to age:

The question of whether a reduced creatinine clearance, or estimated GFR…is normal or abnormal, i.e. disease state or normal for age. There's a philosophical debate that's complicated and unnecessarily polarised in my opinion. (Renal Consultant A)

The clinician dissonance rooted in tensions between perceptions of individual and population approaches to health, conceptions of ‘normal’ and generalism and specialism and also between conflicting philosophical and ethical approaches to biomedicine. GPs experienced feelings of dissonance around two contrasting biomedical ethical frameworks—consequentialism and deontology. These approaches embody different conceptions of moral obligation that can influence clinician decision-making on whether to disclose a diagnosis of CKD.

Most clinicians adopted a (benign) paternalistic approach to disclosure of early-stage CKD that appeared to be underpinned by consequentialism where the outcome or consequences of disclosure of CKD is considered the important factor. The logic of this approach being: If very few people progress from a chronic condition to established renal failure, can it be called a disease? If not, why disclose, label, monitor and potentially alarm people with mildly reduced kidney function?

People have published papers saying a great proportion of people with CKD don't know about it. That may actually be just fine because the GPs may be playing a good game and saying I'm not going to bother this patient with having a GFR of 59 because I know that although it qualifies as CKD 3 it's not gonna make any difference to how I manage that patient and I think that's good medicine rather than, you know, dumping a disease label. I'm sure that you can harm people by saying you've got chronic kidney disease and it's stage 3 out of 5 …and walk away and I'm sure people have been harmed by that. (Renal Consultant B)

In contrast, some, more recently qualified GPs, appeared to adopt a deontological approach to disclosure. In this moral framework, the rights of patients, respect for patient autonomy and shared decision-making practices are likely to be the driving factors in decision-making on disclosure of CKD.

My personal policy I would always disclose…generally speaking I would always explain the diagnosis. I always say it's er, it's a horrible name for this condition and it's not, you know it's misleading and I try and make less of a big deal about it…(GPG)

Although some GPs preferred to disclose a diagnosis of early-stage CKD, they still felt ambivalent about the term CKD and how being labelled as having ‘chronic kidney disease’ was potentially misleading for patients with non-progressive, early-stage CKD.

### Identifying agreement: clinician consonance

#### The meaning of renal decline

Clinicians generally agreed that there is a mismatch between the term ‘chronic kidney disease’ and the ontology of renal decline. Most clinicians conceptualised the broader physiological meaning and clinical implications of reduced kidney function as primarily a barometer of physiological challenge and quality of care.

I would argue that kidney function gives us a barometer on the quality of clinical care and the extent of clinical challenge or metabolic challenge or physiological challenge … So the kidney tells you about the path of physiological challenge… If you measure the protection of the kidney then it tells you about the quality of…medical clinical care. (Renal Consultant C)

The majority of clinicians understood the ontology and meaning of renal impairment as an ‘epiphenomenon’ of other diseases rather than a discrete disease entity.

Is it more an epiphenomenon of their other diseases, their hypertension and their diabetes rather than being an unpleasant independent nasty causative pathology?…So, yes, that is true, ok—you're not gonna do anything renal specific—but in fact for a lot of the common conditions that cause CKD i.e. diabetes, hypertension, vascular disease, you're not practically gonna be doing anything very specialised in renal clinics than you are gonna do in GP clinics. (Renal Consultant A)

I think with CKD it's always going to be secondary prevention [of other diseases]…Nor would I know whether the effort targeted at CKD would be more effective than having the same effort targeted at hypertension and diabetes. (Public Health Physician B)

Clinicians mostly agreed the main purpose of measuring kidney function was in the context of comorbidities such as diabetes and cardiovascular disease. Thus, they argued that the management of CKD should be part of the management of those primary conditions.

Clinicians agreed that renal function should not be assessed in isolation and should be considered in the context of the whole patient. Given this understanding of kidney health, clinicians explained how a holistic approach to the assessment and management of kidney function would be more appropriate than a renal specific one.

What we need to do is to have a holistic approach… It will tell you about whether or not you've spoken to somebody and you've treated them with dignity and all of that but I would equally argue that you can't actually assess somebody and manage them really well if at the same time you're treating them like, you know, a piece of meat, you know, you can't actually do that assessment…to separate out that. If you're just looking at the kidney function, or the blood pressure, or, you know, the temperature or if you're looking at just one parameter you can fall into the trap of treating pneumonia as opposed to treating the person…If you did that for the kidney you'd fail cos there's kind of so many elements you need to do….All the key elements, you know, come together. (Renal Consultant D)

This extract draws attention to how the concept of CKD is seen by clinicians to belie the actual complexity and irreducibility of kidney health to a simple disease construct.

#### How to improve identification of renal pathology

Clinicians in our study agreed that identification of CKD via measurement of eGFR within the current classification system (1–5) was a ‘blunt’ instrument for detecting CKD.

Well, we see any awful lot of elderly people with CKD's just below 60 [eGFR] who have been like that for about seven, eight years…And they've not progressed and I just wondered if you know, if there was perhaps better information about who really need to follow through it because I think some people seem to have CKD but never seem to progress and obviously some people do and how you identify those people. Who we should be concentrating on and perhaps calling it everybody who's got a CKD of 58 in every year to be monitored…hopefully there'll sooner be more information about who will be better to monitor rather than seeing and monitoring everybody. (Practice Nurse B)

Most clinicians agreed that the additional detection of proteinuria at all CKD stages was important for identifying people who were at risk of progression and further morbidity.

Well proteinuria, irrelevant of—you know the stage—the staging system is really defined but you know it's not great, but proteinuria is a stronger marker of poor outcome be that vascular events or progression to end stage renal failure or Acute Kidney Injury…proteinuria's been known for you know from ancient times and could be related to poor outcomes—you know we just know that even teeny weeny bits of proteinuria are generally bad for you predominantly from the vascular perspective to an extent and kidney perspective, so it's moving from a dialysis-centric approach to a health paradigm where the kidneys are about vascular health. (Renal Consultant D)

There are patients who you are measuring and they've got a level of CKD and they're 95 and you can quite honestly say that they are not going to progress because they have no proteinuria, they are unlikely to progress within their normal lifespan …CKD 1 or 2 in a young person with proteinuria may we be very relevant because they've got plenty of years to progress but that might not be so relevant in someone that's 70, 80…So I think it's very hard to make a gross generalisation on it and I think you do have to believe in each individual. (GPP)

However, clinicians felt that the current 2008 NICE guidance^24^ did not encourage proteinuria testing at CKD stages 1 and 2:

Proteinuria is a different matter but we are really not encouraged to do the proteinuria until they are in to CKD 3…We can do it, sure if we were encouraged to do it before then. (GPF)

Some clinicians hoped that a more effective and cheaper method of detection could be developed that would identify patients who would benefit from early intervention:

It would be fantastic to have some way of better predicting which of these people are going to have problems. (GPD)

Clinicians agreed that kidney function was an indicator of vascular health. From this standpoint, clinicians argued for improved identification of CKD in patients who would benefit from early intervention, making early-stage CKD more meaningful for patients and clinicians. Improved identification of early-stage CKD would also help to better target National Health Service (NHS) resources in primary care.

## Discussion

In understanding the tensions around the management of early-stage CKD in primary care and in the context of English national guidelines and QOF, we identified an overarching theme of cognitive dissonance rooted in the logic of contrasting models of healthcare and ethical decision-making. The inclusion of the words ‘chronic’ and ‘disease’ in the term CKD was also identified as a source of tension for clinicians. This term appears to be too simplistic to describe a medical construct that primarily relates to increased risk of morbidity, particularly cardiovascular morbidity.

However, despite the issues of contention and dissonance around early-stage CKD monitoring in primary care, our study found significant agreement between generalist and specialist clinicians' regarding categorising people as having CKD on the basis of reduced eGFRs alone. Clinicians perceived the current classification guidelines as oversimplifying a complex picture of interdependent health and demographic factors, posing ethical dilemmas around diagnosis and disclosure. Given the multilayered nature of the CKD construct and overinclusive methods of identification, based on national guidance at the time,^23^ it is not surprising that the purpose of monitoring early-stage CKD in primary care seemed unclear and there was tension around disclosing a potentially frightening diagnosis to patients.^15^ ^25^ Clinicians agreed the need for improved detection of progressive renal disease and a move towards a patient-centred, holistic assessment with renal function being viewed as a marker of ‘well-being’ and vascular health.

### Strengths and weaknesses of the study

This in-depth qualitative study on clinicians’ experiences of managing early-stage CKD included a range of health professionals. Purposive sampling for maximum variation across service sectors allowed us to examine a broad range of clinician experiences and perspectives on early-stage CKD monitoring in primary care. The achievement of data saturation together with the rigour of analysis increases the credibility of findings. The majority of interviews were conducted before the publication in July 2014 of updated NICE clinical guidelines for CKD and the results should be viewed in that light.^26^

### Comparison with other studies

Our findings of tensions around early-stage CKD monitoring in primary care built upon findings from existing qualitative studies on understanding the management of CKD.^13^ ^15^ ^16^ Our study has deepened the understanding by articulating how different ethical approaches to biomedicine and a preventative medicine agenda created clinician tensions around the efficacy of clinical guidelines for early-stage CKD monitoring. Our findings are consistent with existing literature: GPs found diagnosing CKD via eGFR tests a ‘hit-and-miss’ affair and reported feeling anxious about telling patients with early-stage CKD of their diagnosis.^13^ ^15^ ^23^ Recent research informed by the principles of ‘minimally disruptive medicine’^27^ has addressed the concerns expressed by our participants and demonstrated that it is possible to provide information about CKD without having a detrimental effect on patient anxiety.^28^

Reflecting the ethical dilemmas reported by clinicians in our findings, a kidney-related study in clinical ethics^29^ found that although doctors have a duty to disclose relevant truths to their patients, it is not always clear what constitutes the ‘truth’. The moral obligation to avoid harm may outweigh the doctor's duty to fully disclose the ‘truth’ where the meaning is unclear, as was the case for clinicians in our study. Our findings provide an understanding of why clinical guidelines are not always adhered to for virtuous and complex reasons.

### Implication for clinicians and policymakers

The updated 2014 NICE clinical guidelines for CKD^26^ include recommendations to increase proteinuria testing and not to determine the management of CKD solely by the age of the patient, which addresses some of the concerns of GPs reflected in our study. Anchoring the meaning and purpose of CKD monitoring by increased proteinuria testing and assessment of risk, on an individual patient basis, may help to allay some of the ethical and moral reservations clinicians expressed around the medicalisation of ‘normality’ and harm to patients in being given a disease label.

As national policy moves towards the use of renal markers that are more closely associated with cardiovascular risk, such as cystatin C,^30^ the link between a CKD diagnosis and true increased cardiovascular risk will become more accurate. In turn, this may improve the implementation of guidance as GPs experience increased congruity with the strategic and philosophical aims of diagnosis and monitoring of CKD in primary care.

### Unanswered questions and future research

We do not yet know how the new NICE guidance will be implemented, and how GPs will respond to the introduction of another eGFR formula (CKD-EPI) which will alter CKD diagnoses and stages for some patients,^26^ although this may lead to some resolution of clinician dissonance observed in this study. This may present a positive step as it will limit ‘overdiagnosis’ of CKD and therefore reduce concerns expressed by our participants. However, as the ability to determine true cardiovascular risk becomes more accurate within the cohort of patients currently identified as having CKD, there will be a need for increased understanding around how to translate that biomedical knowledge into improved diagnostic and monitoring strategies, which have clear value to GP and patient.
